# Temporal trends and factors associated with live preterm births in Espírito Santo, 2012-2021

**DOI:** 10.1590/1980-549720260008

**Published:** 2026-03-16

**Authors:** Marcone Marques da Rocha, Laércio da Silva Paiva, Lara Bourguignon Lopes, Raiza Brito Cipriano, Lucca Tamara Alves Carretta, Fernando Rocha Oliveira

**Affiliations:** I Escola Superior de Ciências da Santa Casa de Misericórdia de Vitória, Graduate Program in Public Policies and Local Development - Vitória (ES), Brazil.; II Centro Universitário FMABC, Department of Collective Health - Santo André (SP), Brazil.; III Escola Superior de Ciências da Santa Casa de Misericórdia de Vitória, Department of Medicine - Vitória (ES), Brazil.

**Keywords:** Infant, Infant, Premature, Maternal health, Infant, Newborn, Diseases, Infant mortality

## Abstract

**Objective:**

To analyze the temporal trend of live premature births in the State of Espírito Santo (Brazil) between 2012 and 2022 and to identify associated factors.

**Methods:**

Ecological study using secondary data from the Live Birth Information System (SINASC), covering premature births in Espírito Santo between 2012 and 2022. Prais-Winsten regression was used to assess the temporal trend of the prematurity rate, and the Prevalence Ratio (PR) was used to identify factors associated with premature birth.

**Results:**

A higher prevalence of premature birth was observed among boys (PR=1.06) with weight <2,500 g, Apgar score at one and five minutes <7 (PR=3.10 and PR=4.05), and with the presence of congenital anomalies (PR=2.74). Among maternal factors, higher prevalences were found among mothers with no schooling (PR=1.51), without a partner (PR=1.11), aged <19 years or >35 years (PR 1.14; PR 1.32), who did not have prenatal care (PR=2.32), twin pregnancy (PR=6.21) and cesarean delivery (PR=1.05). In all analyses, statistical significance was p<0.001. Regarding the temporal trend analysis, Barra de São Francisco, Santa Teresa, and Vitória showed an increasing prematurity rate (2012-2022). Alegre, Cachoeiro de Itapemirim, and Itapemirim showed decreasing rates. Espírito Santo showed a stationary rate.

**Conclusion:**

Espírito Santo has a stationary prematurity rate, with some microregions showing increasing or decreasing rates, indicating territorial heterogeneity and regional inequalities in access to health. Maternal and gestational socioeconomic risk factors stand out as indicators of a higher prevalence of prematurity.

## INTRODUCTION

Prematurity and its associated complications constitute a global problem that significantly affects maternal and child health and represent an important indicator of the quality of obstetric and neonatal care. Preterm births, defined as those occurring before 37 weeks of gestation, are associated with high rates of neonatal morbidity and mortality, as well as long-term developmental impairments^1^ ^,^ ^2^.

In Brazil, the analysis of temporal trends in prematurity and its associated factors in specific local contexts, such as the state of Espírito Santo (ES), is essential for addressing health inequalities and improving maternal and infant outcomes. Socioeconomic, demographic, and health service access factors may influence prematurity rates, and the identification of these factors can inform public health actions and intervention strategies^3^ ^,^ ^4^.

In this context, the present study aims to analyze, with depth and methodological rigor, the temporal trends of live preterm births in Espírito Santo between 2012 and 2022, as well as the associated factors that shape this complex phenomenon. Through a comprehensive and integrated analysis, this study seeks to contribute to the understanding and management of prematurity at birth, with implications for public health and population well-being.

## METHODS

This ecological study analyzed secondary data on preterm live births (<37 weeks of gestation)^5^ in the state of ES, Brazil, from 2012 to 2022. The data were obtained from the Information System on Live Births (*Sistema de Informações sobre Nascidos Vivos* - SINASC) and included maternal and neonatal characteristics. The state of ES was selected as the study setting due to its population diversity, with an estimated population of 3,833,712 inhabitants according to the Brazilian Institute of Geography and Statistics (*Instituto Brasileiro de Geografia e Estatística* - IBGE)^6^.

SINASC is a national database of the Ministry of Health that is populated through the Live Birth Declaration (*Declaração de Nascido Vivo* - DNV), a mandatory document in Brazil. The system provides standardized information on the mother, pregnancy and delivery, and the newborn. It is a publicly accessible database and is widely used in epidemiological studies because it enables the monitoring of maternal and child health indicators^7^.

Live births are defined as the complete expulsion or extraction of the product of conception from the mother’s body, regardless of gestational age, that, after separation, breathes or shows signs of life, such as a heartbeat, umbilical cord pulsation, or voluntary muscle contraction^8^. The number of preterm live births refers to infants with a gestational age of less than 37 weeks who, after complete expulsion or extraction from the mother’s body, exhibit at least one sign of life^5^ ^,^ ^8^. The prematurity rate was calculated as the proportion of preterm births in relation to the total number of live births during the analyzed period^9^.

The information collected on live births included gender, race/color, birth weight, presence of congenital anomalies, and Apgar scores at 1 and 5 minutes. Maternal sociodemographic variables included education level, marital status, and age. Pregnancy-related information comprised the number of prenatal visits and whether this number was considered adequate (≥6 visits), according to the definition of the Ministry of Health^10^. The type of pregnancy and the type of delivery were also examined.

For statistical analysis, Prais-Winsten regression was used to assess temporal trends in the prematurity rate between 2012 and 2022, stratified by IBGE microregions. The slope coefficient (β), probability value (p), predictive capacity (r^2^), annual percentage change (APC), and mean APC were calculated. Prevalence ratio (PR) analyses were conducted to identify factors associated with preterm birth.

The data analyzed were public and secondary, obtained from the Information System on Live Births of the Department of Informatics of the Unified Health System (*Departamento de Informática do Sistema Único de Saúde* - DATASUS). Therefore, approval by a research ethics committee was not required, in accordance with Resolutions No. 466/2012 and No. 510/2016 of the National Health Council.

## Data availability statement:

The entire dataset supporting the results of this study is available upon request to the corresponding author.

## RESULTS

Among preterm live births, a slight predominance of males over females was observed, and most were classified as Brown, followed by White and Black. The occurrence of congenital anomalies was 2%, and the most prevalent birth weight was ≥2,500 g. Regarding neonatal vitality, 26.7% presented an Apgar score <7 at the first minute of life; however, most recovered by the fifth minute, and 7.6% remained with unsatisfactory scores (Table 1).

**Table 1.** Sociodemographic characteristics of mothers and live births, 2012-2022.

Characteristics	Preterm	Term
	n (%)	
Gender		
Female	27,421 (47)	261,129 (48.9)
Male	30,919 (53)	273,193 (51.1)
Race/color		
White	15,092 (26.5)	137,324 (25.46)
Black	3,856 (6.8)	32,430 (6.01)
Yellow	200 (0.4)	1,551 (0.29)
Brown	37,468 (65.8)	352,775 (65.36)
Indigenous	189 (0.3)	1,410 (0.26)
Birth weight (g)		
≥2,500	28,679 (49.2)	516,109 (96.5)
1,500-2,499	22,435 (38.4)	17,609 (3.3)
500-1,499	4,203 (7.2)	283 (0.1)
<500	3,060 (5.2)	350 (0.1)
Congenital anomaly		
No	56,764 (98.0)	527,968 (99.4)
Yes	1,147 (2.0)	3,163 (0.6)
Apgar score at 1 minute		
≥7	42,234 (73.28)	483,001 (91.25)
<7	15,400 (26.72)	46,342 (8.75)
Apgar score at 5 minutes		
≥7	53,250 (92.38)	522,138 (98.62)
<7	4,392 (7.62)	7,315 (1.38)
Education (years)		
None	169 (0.3)	936 (0.2)
1 to 3	1,115 (1.9)	8,011 (1.5)
4 to 7	10,371 (18.0)	90,979 (17.2)
8 to 11	33,658 (58.6)	321,445 (60.6)
12 or more	12,209 (21.2)	108,714 (20.5)
Marital status		
With partner	31,830 (55.0)	307,447 (58.0)
Without partner	25,991 (45.0)	222,073 (42.0)
Maternal age (years)		
<19	9,286 (15.9)	78,945 (14.8)
20 to 34	37,993 (65.1)	375,498 (70.3)
>35 years	11,100 (19.0)	79,911 (14.9)
Number of prenatal visits		
None	1,203 (2.1)	5,895 (1.1)
1 to 3	6,720 (11.6)	27,340 (5.1)
4 to 6	20,862 (36.0)	117,837 (22.1)
7 or more	29,079 (50.3)	381,142 (72)
Prenatal care adequacy		
Adequate	24,784 (60.6)	291,055 (75.4)
Inadequate	15,519 (37.9)	92,180 (23.9)
No prenatal care	611 (1.5)	2,733 (0.7)
Type of pregnancy		
Singleton	51,221 (87.8)	528,258 (98.9)
Twin	7,131 (12.2)	5,854 (1.1)
Type of delivery		
Vaginal	20,940 (35.9)	199,652 (37.4)
Cesarean section	37,397 (64.1)	334,398 (62.6)

Maternal age among preterm births was predominantly between 20 and 34 years; however, substantial proportions of adolescents (15.9%) and women aged 35 years or older (19.0%) were also observed, groups recognized as having higher obstetric risk. Most mothers had 8 to 11 years of schooling, and the majority had a partner. Regarding prenatal care, only half of the pregnant women attended seven or more consultations, whereas 37.9% had an inadequate number of visits and 1.5% received no prenatal care. Most pregnancies were singleton and resulted in cesarean delivery (Table 1).

Preterm infants were more frequently male than those born at term, more often classified as brown race/color, and presented lower Apgar scores at one and five minutes. Regarding maternal characteristics, higher proportions of mothers aged <19 and >35 years were observed among preterm births compared with term births; similar patterns were found for absence of a partner and lower maternal educational attainment. The frequency of fewer than seven prenatal visits and the absence of prenatal care were also higher among preterm births. Although most pregnancies were singleton in both groups, twin pregnancies were more frequent among preterm births, as was the predominance of cesarean delivery (Table 1).

The results presented in Table 2 highlight factors associated with prematurity. Male gender showed a significant association with prematurity (PR=1.06), indicating a 6% higher probability of preterm birth compared with female gender. Black, Asian, and Indigenous race/color categories presented a higher prevalence of prematurity compared with the White category, whereas Brown race/color showed a lower prevalence. Infants with birth weight <2,500 g exhibited significantly higher PRs. In addition, the presence of congenital anomalies and Apgar scores <7 at one and five minutes were strongly associated with prematurity (Table 2).

**Table 2.** Multivariable analysis of factors associated with prematurity among live births, 2012-2022.

Characteristics	Preterm live births2012-2022	
	PR (95%CI)	p-value
Gender		
Female	1	
Male	1.06 (1.05-1.08)	<0.001
Race/color		
White	1	
Black	1.07 (1.03-1.10)	<0.001
Yellow	1.15 (1.01-1.31)	0.03
Brown	0.96 (0.95-0.98)	<0.001
Indigenous	1.19 (1.04-1.36)	0.01
Birth weight (g)		
≥2,500	1	
1,500-2,499	10.64 (10.49-10.79)	<0.001
500-1,499	17.79 (17.55-18.04)	<0.001
<500	17.04 (16.77-17.32)	<0.001
Congenital anomaly		
No	1	
Yes	2.74 (2.60-2.88)	<0.001
Apgar score at 1 minute		
≥7	1	
<7	3.10 (3.05-3.15)	<0.001
Apgar score at 5 minutes		
≥7	1	
<7	4.05 (3.95-4.15)	<0.001
Maternal education (years)		
12 or more	1	
None	1.51 (1.31-1.74)	<0.001
1 to 3	1.21 (1.14-1.28)	<0.001
4 to 7	1.01 (0.98-1.03)	0.289
8 to 11	0.92 (0.92-0.95)	<0.001
Marital status		
With partner	1	
Without partner	1.11 (1.09-1.13)	<0.001
Maternal age (years)		
20 to 34	1	
<19	1.14 (1.12-1.17)	<0.001
>35	1.32 (1.30-1.35)	<0.001
Number of prenatal visits		
7 or more	1	
None	2.39 (2.26-2.52)	<0.001
1 to 3	2.78 (2.71-2.82)	<0.001
4 to 6	2.12 (2.08-2.15)	<0.001
Prenatal care adequacy		
Adequate	1	
No prenatal care	2.32 (2.16-2.50)	<0.001
Inadequate	1.83 (1.80-1.87)	<0.001
Type of pregnancy		
Singleton	1	
Twin	6.21 (6.10-6.32)	<0.001
Type of delivery		
Vaginal	1	
Cesarean section	1.05 (1.04-1.07)	<0.001

Mothers with no formal schooling showed a higher prevalence of preterm births compared with those with 12 or more years of education; a similar pattern was observed among mothers without a partner compared with those with a partner. Maternal age <19 years or >35 years was associated with a higher prevalence ratio for prematurity, as were twin pregnancies. Regarding prenatal care, the absence of visits and a low number of prenatal visits were also associated with a higher prevalence of prematurity (Table 2).

**Figure 1.**
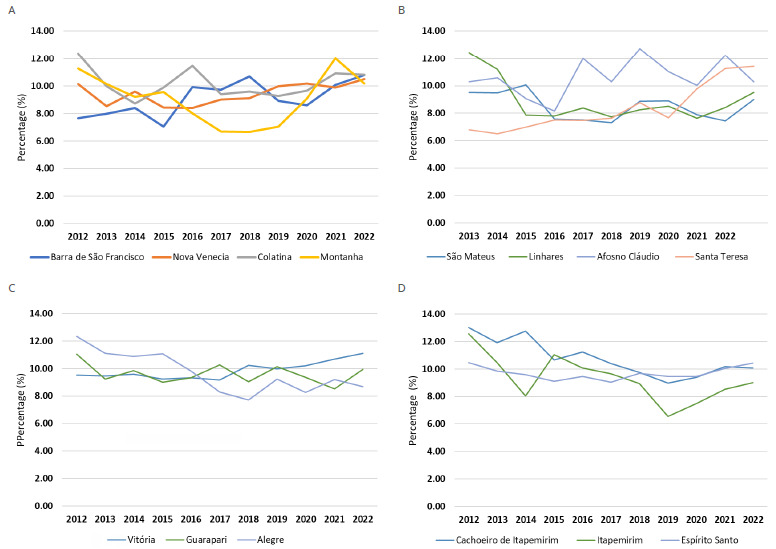
Trend in prematurity rates across microregions of Espírito Santo according to the Brazilian Institute of Geography and Statistics (2012-2022).

**Figure 2.**
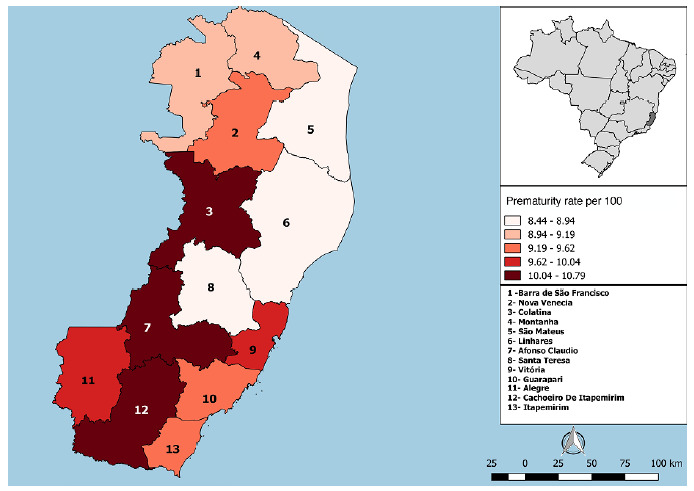
Spatial distribution of prematurity rates in Espírito Santo from 2012 to 2022.

Table 3 presents the historical trends in prematurity across the microregions of ES, while Figure 1 illustrates temporal variations over the study period, and Figure 2 provides a spatial perspective, allowing visualization of the geographic distribution of prematurity within the state. Between 2012 and 2022, the overall prematurity rate in ES remained stationary, although regional variations were observed.

**Table 3.** Trend analysis of the prematurity rate by Brazilian Institute of Geography and Statistics microregion, 2012-2022.

Total period (2012-2022)	APC (95%CI)	r²	p-value	Trend
Barra de São Francisco	2.33 (0.69-4.71)	0.517	0.015	Increasing
Nova Venecia	0.93 (-0.69-2.33)	0.610	0.235	Stationary
Colatina	0.003 (-2.28-2.33)	0.559	0.666	Stationary
Montanha	-0.69 (-6.67-4.71)	0.561	0.764	Stationary
São Mateus	-1.14 (-2.28-1.62)	0.460	0.329	Stationary
Linhares	-2.28 (-6.67-2.33)	0.71	0.261	Stationary
Afonso Claúdio	1.39 (-0.69-2.33)	0.474	0.150	Stationary
Santa Teresa	4.71 (2.33-7.15)	0.695	0.001	Increasing
Vitoria	1.39 (0.23-2.33)	0.865	0.013	Increasing
Guarapari	-0.46 (-1.83-0.46)	-	0.238	Stationary
Alegre	-2.28 (-4.5 to -0.69)	0.827	0.012	Decreasing
Cachoeiro de Itapemirim	-2.73 (-4.5 to -0.92)	0.809	0.005	Decreasing
Itapemirim	-3.39 (-6.67-0.18)	0.552	0.044	Decreasing
Espírito santo	0.05 (-1.6-1.62)	0.915	0.953	Stationary

Some microregions, such as Barra de São Francisco, Santa Teresa, and Vitória, exhibited increasing trends in the proportion of preterm births, indicating a consistent rise throughout the historical series. In contrast, microregions such as Alegre, Cachoeiro de Itapemirim, and Itapemirim demonstrated decreasing trends. The remaining microregions showed stationary behavior, with no significant variations over the period, revealing a heterogeneous pattern within the state (Table 3; Figures 1 and 2).

## DISCUSSION

The findings of this study indicate that prematurity is strongly associated with a set of biological and social factors, reinforcing its multifactorial nature^11^ ^,^ ^12^. Associations with maternal characteristics further support this perspective: the higher prevalence among mothers with lower levels of education reflects barriers to access to information and health services, while the absence of a partner suggests greater social vulnerability and reduced support during pregnancy. In addition, the higher frequency of prematurity at the extremes of maternal age reflects the influence of structural inequalities on maternal and child health and underscores the relevance of public policies targeting populations at increased risk^13^ ^,^ ^14^ ^,^ ^15^.

The gender distribution among preterm infants showed a predominance of males, corroborating findings reported in previous studies^11^ ^,^ ^16^. Slower development of the lungs and immune system in males, as well as the protective effect of higher estrogen levels in females, has been described as a possible explanation for this pattern^17^ ^,^ ^18^. An unequal distribution of prematurity was also observed across race/color categories, with higher prevalences among non-White groups. This pattern may be related to socioeconomic conditions, access to health services, and the quality of prenatal care^19^ ^,^ ^20^. Additionally, structural racism has been identified as a factor that limits access to resources and opportunities, thereby exacerbating health disparities^21^.

Preterm infants showed a significantly higher prevalence of birth weight below 2,500 g. Considering that newborns typically reach this weight at approximately 37 weeks of gestation, when they are classified as full term, this finding is expected in the context of prematurity. For a more accurate assessment of fetal growth restriction, the use of indicators such as weight-for-gestational-age *Z*-scores is required, as these measures can distinguish whether low birth weight results exclusively from prematurity or from intrauterine growth restriction^22^ ^,^ ^23^.

An additional relevant finding was the association between prematurity and congenital anomalies. Although most infants did not present congenital anomalies, their prevalence was higher among preterm infants, consistent with reports in the literature^24^ ^,^ ^25^. This relationship may be associated with genetic, teratogenic, and environmental factors that affect fetal development. In certain situations, the presence of an anomaly may precipitate delivery due to clinical complications that trigger spontaneous labor or require early termination of pregnancy because of maternal-fetal risk^24^ ^,^ ^25^ ^,^ ^26^.

Apgar scores in the first minutes after birth are an important indicator of neonatal health status. Preterm infants presented a higher proportion of Apgar scores lower than 7 at one and five minutes compared with term infants, underscoring their greater physiological vulnerability. These differences indicate that preterm infants require more intensive immediate support and specialized neonatal care, which is associated with longer hospital stays, increased healthcare costs, and a higher risk of neonatal morbidity and mortality^12^ ^,^ ^27^.

The higher frequency of cesarean deliveries and twin pregnancies among preterm births reflects the greater clinical complexity of these pregnancies and the need for intensified obstetric monitoring^28^. The significantly higher prevalence of cesarean sections can be explained by their indication in the presence of complications such as acute fetal distress and intrauterine growth restriction, conditions that often culminate in preterm delivery^29^. In twin pregnancies, increased maternal physiological burden contributes to complications such as preterm labor. Additionally, twin gestations are more frequently associated with obstetric indications for early termination of pregnancy^30^.

The Rio de Janeiro State Federation of Industries (*Federação das Indústrias do Estado do Rio de Janeiro* - FIRJAN) Municipal Development Index (*Índice FIRJAN de Desenvolvimento Municipal* - IFDM) assesses the level of socioeconomic development of Brazilian municipalities. In the health dimension, the IFDM incorporates indicators related to prenatal care coverage, infant mortality from preventable causes, and hospitalizations sensitive to primary care. ES was classified as having a moderate level of health development; however, only 1.3% of its municipalities reached a high level, while nearly 40% remained at a low level^31^.

These disparities reflect substantial heterogeneity among municipalities and the specific characteristics of local health care networks, which influence the regional variability in prematurity rates observed in this study^32^ ^,^ ^33^. The microregions of Barra de São Francisco and Santa Teresa, characterized by a predominantly rural profile and municipalities concentrated in areas of moderate or low development, exhibited increasing trends in prematurity. This pattern may be related to barriers in access to adequate prenatal care and delays in the timely referral of high-risk pregnant women^34^.

In Vitória, despite a high IFDM classification, the observed increase in prematurity rates may be related to the concentration of high-complexity maternity hospitals that receive high-risk pregnant women from across the state^35^. Conversely, microregions in southern Espírito Santo exhibited decreasing trends, which may be associated with the presence of well-structured regional hospitals and greater integration between primary care and referral services. This pattern is consistent with improvements in the health dimension of the IFDM in these municipalities; in Cachoeiro de Itapemirim, for instance, the index increased from 0.56 in 2013 to 0.70 in 2023^34^.

Given the persistent challenges in reducing prematurity rates in Brazil, examining the quality of access to health services is particularly relevant, especially when gestational risk stratification is considered a central strategy for preventing adverse outcomes. Adequate identification of high-risk pregnant women, even within primary care, enables timely referral to specialized outpatient services, ensuring multidisciplinary follow-up and targeted interventions. Failures in these processes, whether due to underreporting of risk or barriers to accessing referral services, increase maternal vulnerability and contribute to the persistence of high rates of preterm live births^12^ ^,^ ^36^.

In this context, health education plays a central role by expanding the knowledge of pregnant women and their families regarding warning signs and the importance of adherence to prenatal care. When combined with continuous professional training and improvements in microprocesses within health services, it promotes comprehensive and continuous care. Therefore, investment in actions that strengthen individual care, while also prioritizing health network structuring and equity in access, may represent a promising approach to addressing persistently high rates of preterm births^12^ ^,^ ^33^ ^,^ ^36^.

The results of this study indicate that the rate of preterm live births in ES between 2012 and 2022 remained stable, despite significant regional variations that reflect distinct local contexts of vulnerability. A higher prevalence of prematurity was observed among male newborns; those classified as Black, Asian, or Indigenous race/ethnicity; infants with unsatisfactory Apgar scores; and those with congenital anomalies. Higher prevalences were also associated with lower maternal educational attainment, absence of a partner, extremes of maternal age, a reduced number of prenatal visits, and increased frequencies of twin pregnancies and cesarean deliveries.

These findings reinforce the multifactorial nature of prematurity and underscore the need for strategies that expand access to and improve the quality of prenatal care, while ensuring equity in maternal and child health. Investment in public health actions and educational programs for pregnant women plays an important role, and public policies should prioritize support for pregnant women in vulnerable situations and promote collaboration between the health and education sectors to address preventive actions in a comprehensive and coordinated manner.

This study has some limitations. The ecological study design does not allow for the inference of individual-level causality. The use of secondary data from SINASC may be subject to underreporting and incomplete data entry and does not allow for more robust analyses of associated factors, such as hierarchical modeling, in addition to the absence of relevant variables, including the quality of prenatal care. Furthermore, regional differences in data collection and recording practices may have influenced the results. Finally, contextual factors, such as local policies and broader social determinants, were not directly captured.
