# Supplementary material for: Temporal trends and factors associated with live preterm births in Espírito Santo, 2012-2021
Source: Rev Bras Epidemiol. 2026 Mar 16;29:e260008. doi: 10.1590/1980-549720260008 (PMC12995338; doi:10.1590/1980-549720260008)
Supplement: Translation

# Translation: Tendência temporal e fatores associados ao nascimento de crianças pré-termo vivas no Espírito Santo, 2012-2022

**DOI:** 10.1590/1980-549720260008.2

**Authors:** Marcone Marques da Rocha, Laércio da Silva Paiva, Lara Bourguignon Lopes, Raiza Brito Cipriano, Lucca Tamara Alves Carretta, Fernando Rocha Oliveira

**Affiliations:** I Escola Superior de Ciências da Santa Casa de Misericórdia de Vitória, Programa de Pós-Graduação em Políticas Públicas e Desenvolvimento Local - Vitória (ES), Brasil.; II Centro Universitário FMABC, Departamento de Saúde da Coletividade - Santo André (SP), Brasil.; III Escola Superior de Ciências da Santa Casa de Misericórdia de Vitória, Departamento de Medicina - Vitória (ES), Brasil.

**Keywords:** Lactente, Recém-nascido prematuro, Saúde materna, Doenças do recém-nascido, Mortalidade infantil

## Abstract

**Objetivo:**

Analisar a tendência temporal dos nascimentos prematuros vivos no estado do Espírito Santo (Brasil) entre 2012 e 2022 e identificar os fatores associados.

**Métodos:**

Estudo ecológico com dados secundários do Sistema de Informações sobre Nascidos Vivos (SINASC), abrangendo partos prematuros no Espírito Santo entre 2012 e 2022. Utilizaram-se a regressão de Prais-Winsten na avaliação da tendência temporal da taxa de prematuridade e a razão de prevalência (RP) para identificar fatores associados ao nascimento prematuro.

**Resultados:**

Observou-se maior prevalência de nascimento prematuro entre os meninos (RP=1,06) com peso <2.500 g, Apgar no primeiro e quinto minuto <7 (RP=3,10 e RP=4,05) e a presença de anomalias congênitas (RP=2,74). Entre os fatores maternos, maiores prevalências estavam entre mães sem nenhuma escolaridade (RP=1,51), sem parceiro (RP=1,11), de idade <19 anos ou >35 anos (RP=1,14; RP=1,32), que não realizaram consulta pré-natal (RP=2,32), gestação gemelar (RP=6,21) e parto cesáreo (RP=1,05). Em todas as análises a significância estatística foi de p<0,001. Quanto à análise de tendência temporal, Barra de São Francisco Santa Teresa e Vitória apresentaram taxa de prematuridade crescente (2012-2022). Alegre, Cachoeiro de Itapemirim e Itapemirim apresentaram taxas decrescentes. O Espírito Santo apresentou taxa estacionária.

**Conclusão:**

O Espírito Santo possui taxa de prematuridade estacionária, com algumas microrregiões com taxas crescentes ou decrescentes, indicando heterogeneidade territorial e desigualdades regionais no acesso à saúde. Destacam-se os fatores socioeconômicos maternos e gestacionais de risco como indicadores de maior prevalência de prematuridade.

## INTRODUÇÃO

A prematuridade e suas complicações são um problema global que afeta significativamente a saúde materno-infantil e representa um importante indicador de qualidade dos cuidados obstétricos e neonatais. Os nascimentos pré-termo, definidos como aqueles que ocorrem antes de 37 semanas de gestação, estão associados a altas taxas de morbidade e mortalidade neonatal, além de prejuízos em longo prazo no desenvolvimento^1^ ^,^ ^2^.

No Brasil, a análise das tendências temporais da prematuridade e os fatores associados a ela em contextos locais específicos, como no estado do Espírito Santo (ES), é essencial para abordar as desigualdades em saúde e melhorar os desfechos materno-infantis. Fatores socioeconômicos, demográficos e de acesso aos serviços de saúde podem influenciar as taxas de prematuridade, e a identificação desses fatores pode direcionar ações de saúde pública e estratégias de intervenção^3^ ^,^ ^4^.

Neste contexto, o presente estudo tem como objetivo analisar, com profundidade e rigor metodológico, as tendências temporais dos nascimentos de pré-termos vivos no Espírito Santo do período entre 2012 e 2022 e os fatores associados que moldam esse fenômeno complexo. Por meio de uma análise abrangente e integral, este estudo almeja contribuir para a compreensão e abordagem da prematuridade no nascimento, com implicações de longo alcance para a saúde pública e o bem-estar da população.

## MÉTODOS

Este estudo ecológico analisou dados secundários sobre nascidos-vivos prematuros (<37 semanas de gestação)^5^ no estado do Espírito Santo, Brasil, entre 2012 e 2022. As informações foram obtidas do Sistema de Informações sobre Nascidos Vivos (SINASC), abrangendo características maternas e neonatais. O estado do Espírito Santo foi escolhido como cenário de pesquisa por sua diversidade populacional, estimada em 3.833.712 habitantes segundo o Instituto Brasileiro de Geografia e Estatística (IBGE)^6^.

O SINASC é uma base nacional do Ministério da Saúde alimentada pela Declaração de Nascido Vivo (DNV), documento de preenchimento obrigatório no Brasil. O sistema disponibiliza informações padronizadas sobre a mãe, sobre a gestação e o parto e sobre o recém-nascido. Trata-se de um banco de dados de acesso público amplamente utilizado em estudos epidemiológicos por permitir o monitoramento de indicadores materno-infantis^7^.

Os nascidos vivos são classificados como a expulsão ou extração completa do produto da concepção do corpo da mãe, independentemente da duração da gestação, e que, após essa separação, respira ou manifesta sinal de vida, tal como batimento cardíaco, pulsação do cordão umbilical ou contração voluntária^8^. O número de nascidos-vivos prematuros refere-se ao número de bebês com idade gestacional inferior a 37 semanas que, depois de ser completamente expulso ou extraído do corpo da mãe, exibe algum sinal de vida^5^ ^,^ ^8^. A taxa de prematuridade foi calculada pela proporção de nascimentos prematuros em relação ao total de nascidos vivos no período analisado^9^.

As informações coletadas relacionadas ao nascidos vivos foram: sexo, raça/cor, peso ao nascer, anomalia congênita e Apgar no 1º e 5º minuto. Os dados relacionados às características sociodemográficas maternas foram: escolaridade, estado civil e idade. As informações relacionadas à gestação incluíram número de consultas pré-natal e se a quantidade de consultas foi adequada (≥6 consultas), segundo a definição do Ministério da Saúde^10^. Também se investigaram o tipo de gravidez e o tipo de parto.

Para a análise estatística, utilizou-se a regressão de Prais-Winsten na avaliação da tendência temporal da taxa de prematuridade entre 2012 e 2022, estratificada por microrregiões do IBGE. Foram calculados o coeficiente angular (β), o valor de probabilidade (p), a capacidade preditiva (r²), a mudança percentual anual (APC) e a média da mudança percentual anual (APC). Análises de razão de prevalência (RP) foram realizadas para identificar fatores associados ao nascimento prematuro.

Os dados utilizados são públicos e secundários, provenientes do Sistema de Informação sobre Nascidos Vivos do Departamento de Informática do Sistema Único de Saúde (DATASUS), não sendo necessária a aprovação do comitê de ética e pesquisa, conforme as Resoluções 466/2012 e 510/2016 do Conselho Nacional de Saúde.

## Declaração de disponibilidade de dados:

Todo o conjunto de dados que dá suporte aos resultados deste estudo está disponível mediante solicitação ao autor correspondente.

## RESULTADOS

Entre os nascidos vivos prematuros, observou-se discreta predominância do sexo masculino em relação ao feminino, e a raça/cor da maioria foi registrada como parda, seguida por brancos e pretos. A ocorrências de anomalias congênitas foi de 2% e o peso ao nascer ≥2,500 g. No que tange às condições de vitalidade, 26,7% apresentaram Apgar <7 no primeiro minuto de vida, embora a maioria tenha alcançado recuperação no quinto minuto, e 7,6% permaneceram com escores insatisfatórios (Tabela 1).

**Tabela 1.** Características sociodemográficas das mães e dos nascidos vivos de 2012-2022.

Variáveis	Prematuros	Termo
	n (%)	
Sexo		
Feminino	27.421 (47)	261.129 (48,9)
Masculino	30.919 (53)	273.193 (51,1)
Raça/cor		
Branco	15.092 (26,5)	137.324 (25,46)
Preto	3.856 (6,8)	32.430 (6,01)
Amarela	200 (0,4)	1.551 (0,29)
Pardo	37.468 (65,8)	352.775 (65,36)
Indígena	189 (0,3)	1.410 (0,26)
Peso ao Nascer (g)		
≥2.500	28.679 (49,2)	516.109 (96,5)
1.500-2.499	22.435 (38,4)	17.609 (3,3)
500-1.499	4.203 (7,2)	283 (0,1)
<500	3.060 (5,2)	350 (0,1)
Anomalia congênita		
Não	56.764 (98,0)	527.968 (99,4)
Sim	1.147 (2,0)	3.163 (0,6)
Apgar 1º minuto		
Maior ou igual a 7	42.234 (73,28)	483.001 (91,25)
Menor que 7	15.400 (26,72)	46.342 (8,75)
Apgar 5º minuto		
Maior ou igual a 7	53.250 (92,38)	522.138 (98,62)
Menor que 7	4.392 (7,62)	7.315 (1,38)
Escolaridade (anos)		
Nenhuma	169 (0,3)	936 (0,2)
1 a 3	1.115 (1,9)	8.011 (1,5)
4 a 7	10.371 (18,0)	90.979 (17,2)
8 a 11	33.658 (58,6)	321.445 (60,6)
12 ou mais	12.209 (21,2)	108.714 (20,5)
Estado Civil		
Com parceiro	31.830 (55,0)	307.447 (58,0)
Sem parceiro	25.991 (45,0)	222.073 (42,0)
Idade da Mãe (anos)		
<19	9.286 (15,9)	78.945 (14,8)
20 a 34	37.993 (65,1)	375.498 (70,3)
>35 anos	11.100 (19,0)	79.911 (14,9)
N° Consulta pré-natal		
Nenhuma	1.203 (2,1)	5.895 (1,1)
1 a 3	6.720 (11,6)	27.340 (5,1)
4 a 6	20.862 (36,0)	117.837 (22,1)
7 ou mais	29.079 (50,3)	381.142 (72)
Consulta pré-natal		
Adequada	24.784 (60,6)	291.055 (75,4)
Inadequada	15.519 (37,9)	92.180 (23,9)
Não fez	611 (1,5)	2.733 (0,7)
Tipo de gravidez		
Única	51.221 (87,8)	528.258 (98,9)
Gemelar	7.131 (12,2)	5.854 (1,1)
Tipo de Parto		
Vaginal	20.940 (35,9)	199.652 (37,4)
Cesária	37.397 (64,1)	334.398 (62,6)

A idade materna nos prematuros concentrou-se principalmente na idade entre 20 e 34 anos, mas houve proporções expressivas de adolescentes (15,9%) e de mulheres acima de 35 anos (19,0%), grupos reconhecidos como de maior risco obstétrico. A escolaridade da maioria ficou em torno de 8 a 11 anos, e a maior parte possuía companheiro. Com relação ao acompanhamento pré-natal, apenas metade das gestantes realizou sete ou mais consultas, enquanto 37,9% tiveram número de consultas inadequado e 1,5% não realizou qualquer acompanhamento. A maior parte das gestações foi única e com via de parto cesariana (Tabela 1).

Os prematuros apresentaram predominância do sexo masculino em comparação aos nascidos a termo, assim como a cor/raça pardo e menores valores de Apgar no primeiro e quinto minuto. Com relação às características maternas, houve maior proporção de mães com idade <19 anos e >35 anos entre nascidos prematuros comparados aos nascidos a termo, e o padrão repete-se na ausência de companheiro e na menor escolaridade materna. A frequência de consulta pré-natal inferior a sete e a ausência de consultas também foram superiores nos prematuros e, embora a maioria das gestações tenha sido única em ambos os grupos, a ocorrência de gestações gemelares foi mais elevada entre os prematuros, assim como a predominância de cesariana (Tabela 1).

Os resultados da Tabela 2 salientam fatores associados à prematuridade. O sexo masculino apresentou associação significativa com a prematuridade (RP=1,06), indicando probabilidade 6% maior de nascimento prematuro em relação ao sexo feminino. As raças preta, amarela e indígena mostraram maior prevalência de prematuridade em relação à raça branca, enquanto a raça parda obteve menor prevalência. Bebês com peso inferior a 2,500 g obtiveram RP significativamente elevadas; além disso, a presença de anomalias congênitas e Apgar inferior a 7 no primeiro e quinto minuto mostrou-se fortemente associada à prematuridade (Tabela 2).

**Tabela 2.** Análise múltipla dos fatores associados à prematuridade de nascidos vivos de 2012-2022.

Variáveis	Prematuros vivos 2012-2022	
	RP (IC95%)	Valor p
Sexo		
Feminino	1	
Masculino	1,06 (1,05-1,08)	<0,001
Raça/cor		
Branco	1	
Preto	1,07 (1,03-1,10)	<0,001
Amarela	1,15 (1,01-1,31)	0,03
Pardo	0,96 (0,95-0,98)	<0,001
Indígena	1,19 (1,04-1,36)	0,01
Peso ao Nascer (g)		
≥2.500	1	
1.500-2.499	10,64 (10,49-10,79)	<0,001
500-1.499	17,79 (17,55-18,04)	<0,001
<500	17,04 (16,77-17,32)	<0,001
Anomalia congênita		
Não	1	
Sim	2,74 (2,60-2,88)	<0,001
Apgar 1º minuto		
Maior ou igual a 7	1	
Menor que 7	3,10 (3,05-3,15)	<0,001
Apgar 5º minuto		
Maior ou igual a 7	1	
Menor que 7	4,05 (3,95-4,15)	<0,001
Escolaridade materna (anos)		
12 ou mais	1	
Nenhuma	1,51 (1,31-1,74)	<0,001
1 a 3	1,21 (1,14-1,28)	<0,001
4 a 7	1,01 (0,98-1,03)	0,289
8 a 11	0,92 (0,92-0,95)	<0,001
Estado Civil		
Com parceiro	1	
Sem parceiro	1,11 (1,09-1,13)	<0,001
Idade da Mãe (anos)		
20 a 34	1	
<19	1,14 (1,12-1,17)	<0,001
>35	1,32 (1,30-1,35)	<0,001
Nº consultas pré-natal		
7 ou mais	1	
Nenhuma	2,39 (2,26-2,52)	<0,001
1 a 3	2,78 (2,71-2,82)	<0,001
4 a 6	2,12 (2,08-2,15)	<0,001
Consulta pré-natal		
Adequada	1	
Não fez	2,32 (2,16-2,50)	<0,001
Inadequada	1,83 (1,80-1,87)	<0,001
Tipo de gravidez		
Única	1	
Gemelar	6,21 (6,10-6,32)	<0,001
Tipo de Parto		
Vaginal	1	
Cesárea	1,05 (1,04-1,07)	<0,001

Mães sem escolaridade tiveram maior prevalência de bebês prematuros em comparação com mães com 12 anos ou mais de estudo, o que também ocorreu com as mães sem parceiro em comparação a mães com parceiro. As idades maternas <19 anos ou >35 anos apresentaram maior razão de prevalência para prematuridade, assim como a gravidez gemelar. Quanto ao número de consultas pré-natais, aquelas que não realizaram consultas ou fizeram poucas consultas também obtiveram maior prevalência de prematuridade (Tabela 2).

A Tabela 3 representa a tendência histórica da prematuridade nas microrregiões do ES, enquanto a Figura 1 demonstra essa variação ao longo dos anos e a Figura 2 acrescenta a perspectiva espacial, permitindo visualizar como a prematuridade se distribui geograficamente no estado do Espírito Santo. No período de 2012 a 2022, a tendência geral da taxa de prematuridade no Espírito Santo manteve-se estacionária, embora com variações regionais.

**Tabela 3.** Análise de tendência da taxa de prematuridade segundo microrregião do Instituto Brasileiro de Geografia e Estatística de 2012-2022.

Período total (2012-2022)	APC (IC 95%)	r²	Valor p	Tendência
Barra de São Francisco	2,33 (0,69-4,71)	0,517	0,015	Crescente
Nova Venecia	0,93 (-0,69-2,33)	0,610	0,235	Estacionária
Colatina	0,003 (-2,28-2,33)	0,559	0,666	Estacionária
Montanha	-0,69 (-6,67-4,71)	0,561	0,764	Estacionária
São Mateus	-1,14 (-2,28-1,62)	0,460	0,329	Estacionária
Linhares	-2,28 (-6,67-2,33)	0,71	0,261	Estacionária
Afonso Claúdio	1,39 (-0,69-2,33)	0,474	0,150	Estacionária
Santa Teresa	4,71 (2,33-7,15)	0,695	0,001	Crescente
Vitoria	1,39 (0,23-2,33)	0,865	0,013	Crescente
Guarapari	-0,46 (-1,83-0,46)	-	0,238	Estacionária
Alegre	-2,28 (-4,5 a -0,69)	0,827	0,012	Decrescente
Cachoeiro de Itapemirim	-2,73 (-4,5 a -0,92)	0,809	0,005	Decrescente
Itapemirim	-3,39 (-6,67-0,18)	0,552	0,044	Decrescente
Espírito santo	0,05 (-1,6-1,62)	0,915	0,953	Estacionária

**Figura 1.** Tendência da taxa de prematuridade nas microrregiões do Espírito Santo segundo o Instituto Brasileiro de Geografia e Estatística (2012-2022).

**Figura 2.** Distribuição espacial da taxa de prematuridade no Espírito Santo, no período de 2012 a 2022.

Algumas microrregiões, como Barra de São Francisco, Santa Teresa e Vitória, apresentaram tendência crescente no percentual de nascimentos prematuros, indicando aumento consistente ao longo da série histórica. Em contrapartida, microrregiões como Alegre, Cachoeiro de Itapemirim e Itapemirim mostraram tendência decrescente. As demais microrregiões permaneceram com comportamento estacionário, sem variações significativas no período, revelando um quadro heterogêneo dentro do mesmo estado (Tabela 3; Figuras 1 e 2).

## DISCUSSÃO

Os achados deste estudo demonstram que a prematuridade está fortemente associada a um conjunto de fatores biológicos e sociais, reforçando seu caráter multifatorial^11^ ^,^ ^12^. A associação com as variáveis maternas reforça essa ideia - a maior prevalência entre mães de baixa escolaridade reflete barreiras no acesso à informação e aos serviços de saúde, enquanto a ausência de parceiro aponta para maior vulnerabilidade social e menor suporte durante a gestação. Além disso, a maior frequência nos extremos etários reflete a influência das desigualdades estruturais sobre a saúde materno-infantil, destacando a necessidade de políticas públicas voltadas a grupos de risco^13^ ^,^ ^14^ ^,^ ^15^.

A distribuição por sexo entre os prematuros mostrou predominância masculina, corroborando estudos anteriores^11^ ^,^ ^16^. Os pulmões e o sistema imunológico no sexo masculino se desenvolvem mais lentamente, e os níveis mais elevados de estrogênio nas meninas oferecem proteção contra a prematuridade, justificando o achado^17^ ^,^ ^18^. Também se observou distribuição desigual da prematuridade entre as diferentes raças/cores, com prevalências maiores nos grupos raciais não brancos, o que pode estar relacionado a fatores socioeconômicos, acesso aos serviços de saúde e qualidade da assistência pré-natal^19^ ^,^ ^20^. Além disso, o racismo estrutural dificulta o acesso a recursos e oportunidades, agravando a disparidade na saúde^21^.

Os prematuros apresentaram prevalência significativamente maior de peso ao nascer inferior a 2,500 g. Considerando-se que, em geral, o recém-nascido alcança esse peso por volta das 37 semanas de gestação, quando passa a ser classificado como a termo, trata-se de um achado esperado para a prematuridade. Para uma análise mais acurada da restrição de crescimento fetal, faz-se necessária a utilização de indicadores como os escores Z de peso para a idade gestacional, capazes de identificar se o baixo peso resulta exclusivamente da prematuridade ou de restrição do crescimento intrauterino^22^ ^,^ ^23^.

Outro aspecto importante foi a relação da prematuridade com as anomalias congênitas. Embora a maioria dos bebês não apresente tais anomalias, a prevalência dessas condições é maior entre os prematuros, corroborando outros estudos^24^ ^,^ ^25^. Essa associação pode estar relacionada a fatores genéticos, teratogênicos e ambientais que influenciam o desenvolvimento fetal. Ademais, em determinados casos, a presença da anomalia pode precipitar o parto, por complicações clínicas que levam ao desencadeamento espontâneo do trabalho de parto ou pela necessidade de interrupção gestacional precoce diante do risco materno-fetal^24^ ^,^ ^25^ ^,^ ^26^.

Os escores de Apgar nos primeiros minutos após o nascimento também se mostraram um indicador importante do estado de saúde neonatal. Prematuros apresentaram proporção mais elevada de Apgar menor que 7 no primeiro e no quinto minuto em comparação aos bebês a termo, destacando sua maior vulnerabilidade fisiológica. Tais diferenças demonstram que os prematuros demandam maior suporte assistencial imediato e cuidados intensivos especializados, o que acarreta maior tempo de internação hospitalar, elevação dos custos assistenciais e aumento do risco de morbimortalidade neonatal^12^ ^,^ ^27^.

A maior frequência de cesarianas e gemelares entre os prematuros reflete a complexidade clínica dessas gestações e a necessidade de vigilância obstétrica intensificada^28^. A prevalência significativamente maior de partos cesáreos é explicada pela sua indicação diante de intercorrências, como sofrimento fetal agudo e restrição de crescimento intrauterino, situações que frequentemente culminam em parto pré-termo^29^. Já nas gestações gemelares há aumento da sobrecarga materna, que leva à ocorrência de complicações, como o trabalho de parto prematuro. Além disso, gestações gemelares são mais frequentemente acompanhadas de indicações obstétricas para interrupção precoce^30^.

O Índice da Federação das Indústrias do Estado do Rio de Janeiro (FIRJAN) de Desenvolvimento Municipal (IFDM) avalia o nível de desenvolvimento socioeconômico dos municípios brasileiros. No campo da saúde, o IFDM considera a cobertura pré-natal, a mortalidade infantil por causas evitáveis e as internações sensíveis à atenção primária. O Espírito Santo apresentou um desenvolvimento em saúde considerado moderado, mas apenas 1,3% dos municípios do estado alcançou nível alto, enquanto quase 40% permaneceram em nível baixo^31^.

Essas disparidades refletem a grande heterogeneidade entre os municípios e as especificidades das redes de saúde, que impactam as taxas regionais variadas de prematuridade observadas no estudo^32^ ^,^ ^33^. As microrregiões da Barra de São Francisco e Santa Teresa, de perfil rural e com municípios concentrados em faixas de desenvolvimento moderado ou baixo, apresentaram tendência crescente de prematuridade, possivelmente em decorrência de barreiras de acesso ao pré-natal adequado e ao encaminhamento oportuno das gestantes de risco^34^.

Em Vitória, embora o IFDM seja alto, a elevação das taxas pode estar relacionada à concentração de maternidades de alta complexidade que recebem gestantes de risco de todo o estado^35^. Por sua vez, as microrregiões do sul capixaba apresentaram tendência decrescente, que pode estar associada à presença de hospitais regionais estruturados e à maior integração entre a atenção básica e os serviços de referência. Esse resultado reflete a melhora do IFDM-saúde nesses municípios; em Cachoeiro de Itapemirim, por exemplo, o índice passou de 0,56 em 2013 para 0,70 em 2023^34^.

Perante a dificuldade em reduzir as taxas de prematuridade no Brasil, o estudo da qualidade do acesso aos serviços de saúde é relevante, principalmente se considerarmos a estratificação do risco gestacional como estratégia central na prevenção de desfechos adversos. A adequada identificação de gestantes de alto risco, ainda na atenção primária, permite seu encaminhamento oportuno para serviços ambulatoriais especializados, garantindo seguimento multiprofissional e intervenções direcionadas. Falhas nesses processos, seja pela subnotificação do risco, seja pela dificuldade de acesso a serviços de referência, ampliam a vulnerabilidade de gestantes e contribuem para a manutenção de elevadas taxas de nascidos vivos pré-termo^12^ ^,^ ^36^.

Nesse contexto, a educação em saúde assume papel central, pois amplia o conhecimento das gestantes e de suas famílias sobre sinais de risco e sobre a importância da adesão ao pré-natal. Aliada à capacitação contínua dos profissionais e à melhoria dos microprocessos nos serviços de saúde, favorece a integralidade e a continuidade da atenção. Assim, investir em ações que fortaleçam o cuidado individual, sem perder de vista a estruturação da rede e a equidade no acesso, pode representar um caminho promissor para o enfrentamento das persistentes taxas de nascimentos pré-termo^12^ ^,^ ^33^ ^,^ ^36^.

Dessa forma, os resultados deste estudo demonstram que a taxa de nascidos vivos pré-termo no Espírito Santo entre 2012 e 2022 se manteve estável, embora com variações regionais significativas, revelando contextos locais distintos de vulnerabilidade. Observou-se maior prevalência de prematuridade entre recém-nascidos do sexo masculino, de cor/raça preta, amarela e indígena, escores de Apgar insatisfatórios e presença de anomalias congênitas. Destacam-se ainda a menor escolaridade materna, a ausência de parceiro, os extremos etários, o número reduzido de consultas de pré-natal e a maior frequência de gestações gemelares e partos cesáreos.

Tais achados reforçam o caráter multifatorial da prematuridade e apontam a necessidade de estratégias que ampliem o acesso e a qualidade do pré-natal e assegurem a equidade na saúde materno-infantil. Investimentos em programas de saúde pública e educação para gestantes desempenham importante papel, sendo necessário que políticas públicas priorizem o suporte a gestantes vulneráveis e incentivem a colaboração entre os setores de saúde e educação para abordar ações de prevenção de maneira abrangente e coordenada.

Este estudo apresenta algumas limitações. O delineamento ecológico do estudo não permite inferir causalidade individual. O uso de dados secundários do SINASC pode estar sujeito a subnotificações e preenchimentos incompletos e não possibilita uma análise robusta de fatores associados, como a de modelos hierarquizados, além da ausência de variáveis relevantes, como a qualidade da assistência pré-natal. Além disso, diferenças regionais na coleta e registro das informações podem ter influenciado os resultados. Por fim, fatores contextuais, como políticas locais e determinantes sociais, não foram diretamente captados.
